# Trends and Directions in Oats Research under Drought and Salt Stresses: A Bibliometric Analysis (1993–2023)

**DOI:** 10.3390/plants13141902

**Published:** 2024-07-10

**Authors:** Haiyan Huang, Xiangtao Wang, Junqin Li, Yang Gao, Yuting Yang, Rui Wang, Zijun Zhou, Puchang Wang, Yujun Zhang

**Affiliations:** 1 School of Life Sciences, Guizhou Normal University, Guiyang 550025, China; 15186985817@163.com (H.H.); wxt_11@163.com (X.W.); lijq489@nenu.edu.cn (J.L.); yutingyang0509@163.com (Y.Y.); 13118563086@163.com (R.W.); 15085781094@163.com (Z.Z.); 2 School of Karst Science, Guizhou Normal University, Guiyang 550025, China; gyphoebe945@126.com; 3 Guizhou Provincial Institute of Prataculture, Guizhou Academy of Agricultural Sciences, Guiyang 550006, China; zhangyj_92@163.com

**Keywords:** oats, adversity stresses, bibliometric, R, VOSviewer, Citespace

## Abstract

With global climate change leading to increasing intensity and frequency of droughts, as well as the growing problem of soil salinization, these factors significantly affect crop growth, yield, and resilience to adversity. Oats are a cereal widely grown in temperate regions and are rich in nutritive value; however, the scientific literature on the response of oat to drought and salt stress has not yet been analyzed in detail. This study comprehensively analyzed the response of oat to drought stress and salt stress using data from the Web of Science core database and bibliometric methods with R (version4.3.1), VOSviewer (version 1.6.19), and Citespace (version6.3.1.0) software. The number of publications shows an increasing trend in drought stress and salt stress in oat over the past 30 years. In the field of drought-stress research, China, the United States, and Canada lead in terms of literature publication, with the most academic achievements being from China Agricultural University and Canadian Agricultural Food University. The journal with the highest number of published papers is Field Crops Research. Oat research primarily focuses on growth, yield, physiological and biochemical responses, and strategies for improving drought resistance. Screening of drought-tolerant genotypes and transformation of drought-tolerant genes may be key directions for future oat drought research. In the field of salt-stress research, contributions from China, the United States, and India stand out, with the Chinese Academy of Agricultural Sciences and Inner Mongolia Agricultural University producing the most significant research results. The largest number of published articles has been found in the Physiologia Plantarum journal. Current oat salt-stress research primarily covers growth, physiological and biochemical responses, and salt-tolerance mechanisms. It is expected that future oat salt research will focus more on physiological and biochemical responses, as well as gene-editing techniques. Despite achievements under single-stress conditions, combined drought and salt-stress effects on oat remain understudied, necessitating future research on their interaction at various biological levels. The purpose of this study is to provide potential theoretical directions for oat research on drought and salt stress.

## 1. Introduction

Emerging projections on climate change forecast a pronounced escalation in aridification across extensive global territories [1], heralding extreme weather phenomena marked by elevated temperatures, diminished precipitation, and acute water shortages. Such conditions are pivotal in precipitating droughts [2], which significantly undermine agricultural productivity through heightened yield variability and reduced overall production [3]. Simultaneously, soil salinization emerges as an escalating ecological dilemma, with the area afflicted by salt stress enlarging at an annual rate of 1% to 2% [4,5,6]. It is estimated that approximately 900 million hectares worldwide—representing 6% of total land and 20% of arable land—are impacted by salinization [7,8,9]. Soil salinization is a global ecological problem and one of the main factors contributing to land desertification and cropland degradation. Drought and salt stresses are recognized as critical environmental stressors that markedly reduce the yield potential of crops, limit the expanse of arable territory, and pose challenges to the introduction of agricultural species into novel environments [10,11]. Furthermore, extreme climatic conditions, such as droughts, alongside soil salinization, can precipitate a range of physiological, survival, and developmental adaptations in plants. Additionally, these stressors can alter vegetation structure and functionality [12,13]. Consequently, the potential repercussions of severe drought and soil salinization on specific plant species and ecosystems have attracted widespread research attention globally.

Numerous investigations have illustrated that drought and salt stresses negatively impact plant physiology and development [14,15,16]. Drought stress is known to compromise membrane integrity and functionality, hinder photosynthesis and metabolic processes, and diminish nutrient absorption and distribution in plants [15,17]. These alterations result in reduced water availability, stunted growth and development, and lowered yield and quality [10,18,19]. Similarly, salt stress, instigated by elevated salt concentrations, can curtail water utilization, impede the absorption of crucial minerals, cause dehydration, provoke nutrient deficiencies, and inhibit growth [5,20,21]. This stress can further diminish stomatal conductance and evaporation, therefore reducing the efficiency of carbon assimilation through the accumulation of toxic ions [8].

Oats exhibit considerable tolerance to drought and a moderate tolerance to salt stress, positioning them as a promising candidate for enhancing saline-alkali soils in arid regions [22]. Production of oat has shifted to marginal lands that tend to accumulate salt because it is more salt-tolerant than most grain crops. In China, oat is mainly grown in the northern, northwestern, and southwestern regions. This is especially true of the northwestern provinces of Gansu, Xinjiang, and Inner Mongolia. The soils in these regions have high levels of salt and alkaline substances, leading to land degradation and affecting crop growth. These areas are characterized by cold or dry climates. As a result of climate change, the area of cultivated land with salt damage problems is expected to expand and intensify. Improved ruderalis genotypes may play an important role in rehabilitating saline and other degraded soil conditions [23]. Beyond its adaptability, oat is nutritionally beneficial, with oat oil being rich in unsaturated fatty acids, offering health advantages such as reduced risk of heart disease and improved brain function [24]. The soluble fiber β-glucan in oat provides immune-boosting effects and may aid in regulating blood sugar levels [25,26]. Additionally, oat peptides exhibit anti-inflammatory and antioxidant properties [27,28], and oat protein, a valuable source of essential amino acids, supports vegetarian and vegan diets [12]. The anti-tumor attributes of oat also contribute to the prevention of certain cancers and chronic diseases [29,30], making it a significant subject of agronomic and nutritional research. Nonetheless, drought and salt conditions can impede oat germination, growth, and development, adversely affecting quality, yield, and physicochemical properties [22,24]. Despite extensive investigation into the effects of drought and salt stresses on oat, the literature remains fragmented across various databases. This fragmentation poses challenges in identifying growth trends of core ideas within this field and in conducting a comprehensive analysis across different disciplines. Consequently, there is an urgent need for a quantitative literature analysis to cohesively summarize research progress and trends.

Bibliometric analysis stands as a pivotal research methodology that utilizes statistical and mathematical tools for the thorough examination of literature, aiming to attain an exhaustive understanding of a designated topic area. Bibliometric analysis focuses on the qualitative and quantitative evaluation of publications within databases through the application of statistical and computational techniques [31,32,33], which process enables researchers to gauge the number, quality, and interconnectedness of academic works [34]. Moreover, it sheds light on collaborative trends across journals, countries, and organizations, enhancing our understanding of the organizational framework, influence dynamics, and leading contributors in a specific field of study. Additionally, this method plays a crucial role in the ongoing monitoring and analysis of keywords, assisting in the recognition of emerging trends and shifts within a research domain.

While the body of literature on oat is extensively developed, bibliometric analyses specifically targeting oat research within the contexts of drought and salt stresses remain sparse. Consequently, a bibliometric analysis aims to map out global research trends related to oat drought and salt stresses and to suggest novel directions for future investigations. The objectives of this study are multifaceted: (1) to evaluate the contributions of nations, institutions, and scholars to the knowledge base concerning oat’s responses to salt and drought stresses; (2) to delineate research priorities and trajectories; and (3) to trace the evolution of research themes over time with the intention of forecasting future trends in the examination of oat responses to salt and drought stresses.

## 2. Results

### 2.1. Descriptive Statistics of Publications and Main Information

**Figure 1:**
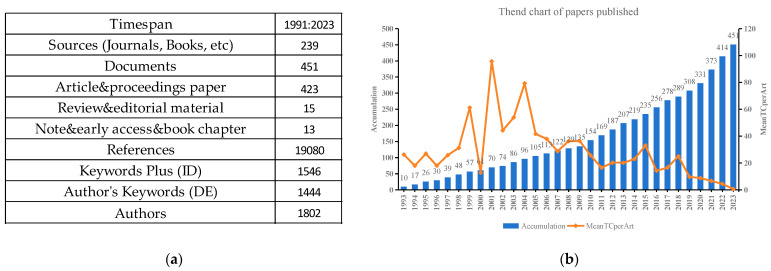
Drought-stress research date of oat from 1993 to 2023, including (**a**) supplementary data of oat drought-stress research; (**b**) The number of papers published between 1993 and 2023; the blue bar indicates the cumulative amount of annual publications; the orange line indicates the average total number of citations per paper (Mean TC per Art).

From 1993 to 2023, a total of 451 papers were published in 239 dissimilar sources on the effects of drought stress on oat. The number of drought-themed articles accounted for 93.8% of these studies, while reviews accounted for 3.3% and book chapters for 2.9% (Figure 1a). Notably, 244 articles, or 54.1% of the total, were published in the last decade, indicating that the field of research on oat response to drought stress has gradually attracted more attention over the past 10 years. The period of study showed a moderate growth rate in publications related to the effects of drought on oat between 1993 and 2012, with a substantial increase observed from 2013 to 2023. The peak annual publication for drought-stress research reached 42 in 2021. A significant shift in citation counts was most notable between 1999 and 2002, achieving the highest mean global total citations at 95.78 in 2001, while the lowest was recorded at 0.65 in 2023 (Figure 1b).

**Figure 2:**
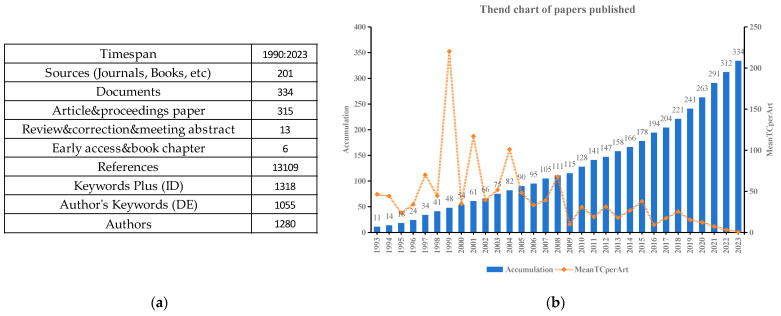
Salt-stress research date of oat from 1993 to 2023, including (**a**) supplementary data of oat salt-stress research; (**b**) the number of papers published between 1993 and 2023, the blue bar indicates the cumulative amount of annual publications; the orange line indicates the average total number of citations per paper (Mean TC per Art).

During the three decades from 1993 to 2023, a total of 334 papers on the effects of salt stress on oat were published in 201 different publications (Figure 2a). These papers were predominantly research articles, which accounted for 94.3% of the total, followed by review articles (3.9%) and book chapters (1.8%). The number of publications concerning oat salt-stress research increased from 11 in 1993 to 334 in 2023, indicating that scientific issues related to this field have attracted increased attention from scientists and society. The average number of citations per publication in oat salt-stress studies found its most substantial change between 1998 and 2000 (Figure 2b). These trends highlight the escalating scholarly interest in the impacts of salt stresses on oat.

Overall, the field of research on oat response to drought and salt stresses has experienced significant growth, with an upward trend in the number of publications. This phenomenon reflects the increasing interest of publishers, regions, institutions, and researchers in this topic, establishing this area as a hotspot for research.

### 2.2. Analysis of the Collaboration Network

#### 2.2.1. Institutions

**Figure 3:**
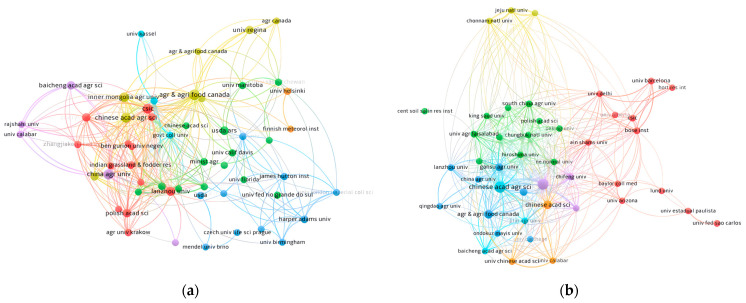
Institutions coupling analysis of oat. (**a**) drought stress (f ≥ 3). (**b**) salt stress (f ≥ 3). Dots indicate the number of papers published by institutions. The larger the node, the more papers published. The width of the connecting line indicates the cooperation frequency between institutions. The thicker the connecting line, the higher the cooperation frequency.

Globally, 585 research institutions and groups are dedicated to exploring the response of oat to drought conditions, while 433 research organizations have delved into the area of salt-stress effects on oat (Figure 3). Figure 3a illustrates the clustering relationships among different institutions, revealing their cooperation status in the field of research on oat response to drought stress. In this clustering analysis, Canadian Agricultural Food University ranked first in terms of node size (Figure 3a), which not only indicates that this institution has the greatest influence in the research field but also reflects its status as a core institution in the field that maintains close and extensive collaborations with other institutions (it has a total connectivity strength of 1215, i.e., TLS). It is followed by China Agricultural University (CAU) and CSS Heavy Industry University (CHIU), which are in second and third place in terms of node size, respectively, two institutions of higher learning that are equally important in academic exchanges. It should be noted that Cranfield University has relatively few partnerships with other institutions despite its position in this academic field.

In the field of research on oat response to salt stress, China Agricultural University and Inner Mongolia Agricultural University, with their large number of publications, have nodes that are particularly significant in the cluster analysis, demonstrating their strong research productivity and extensive academic connections in the field (Figure 3b). Additionally, the Canadian University of Agriculture and Food (CUAF) occupies a significant academic position in this research area and has made notable contributions to research progress. Meanwhile, the Central Soil Salinity Research Institute (CSRI) and Lund University have also contributed to research in this area, but their partnerships and sharing of results with other institutions are relatively weak.

Among the top 10 institutions in terms of the number of papers published in the study of oat response to drought stress, the institutions centered around Canadian Agricultural Food University, China Agricultural University, and CSS Heavy Industry Co. Ltd ranked the top three (Table 1). Among the top 10 institutions, China occupies 60%, and other countries such as the United States, Canada, the United Kingdom, and Poland are also involved, reflecting China’s leading position in the field of research on oat response to drought stress. In terms of the number of published articles, Canadian Agricultural Food University ranked first with 19, followed by China Agricultural University (CAU) with 15 publications. In terms of the number of citations, Canadian Agricultural Food University ranked first with 335, and CSS Heavy Industry Co. Ltd closely followed with 330 citations (Table 1). These data demonstrate the academic influence and contribution of these institutions in the field.

Among the top 10 institutions in terms of publications in the field of salt stress on oat, the China Academy of Agricultural Sciences (CAAS), Inner Mongolia Agricultural University (IMU), and Canadian Agricultural Food University (CAFU) were the top three institutions with 21, 19, and 9 publications, respectively (Table 2). In terms of the number of publications and citations, CAAS led with 21 articles and 215 citations, followed by Inner Mongolia Agricultural University with 19 papers and 187 citations, and Canadian Agricultural Food University with 9 papers and 386 citations. This indicates their outstanding performance in terms of the volume of publications and the strength of inter-institutional collaboration.

Overall, the Canadian Agricultural Food University (CAFU) has been particularly productive in the field of oat response to drought and salt stress, not only publishing numerous papers but also establishing extensive collaborations with several institutions. This highlights its important contribution to this field. Meanwhile, other institutions such as China Agricultural University and the Chinese Academy of Agricultural Sciences (CAAS) also hold important academic positions and maintain close collaborations, further enhancing the research impact in this area.

#### 2.2.2. Countries

The number of scientific research publications and the global citation frequency of a country can reflect the level and focus of a country’s scientific research. From 1993 to 2023, 69 countries studied the response of oat to drought. The results showed that China had the highest number of published and cited papers, with 91 papers accumulating a total of 1211 citations (Table 3). In comparison, the United States ranked second with 63 papers and a total of 2034 citations, and Canada ranked third with 36 papers and 646 total citations. Among the countries with more than 2000 citations, the USA exhibited the highest average number of citations per paper at 32.3, indicating that the USA produces high-quality papers in the field of oat response to drought. These data underscore the significant contributions and the quality of research outputs from these leading countries in addressing the agricultural challenges posed by drought conditions.

In addition, in the field of research on the effects of salt stress on oat, 53 countries around the world have conducted research during the period from 1993 to 2023. The results show that China maintains the highest number of papers, with 97 publications, while the United States (45 papers) and India (33 papers) hold the second and third positions, respectively (Table 4). Among the countries with more than 2000 citations, the average citation frequency of the US was the highest at 51.93, indicating the high quality of US papers on oat response studies under salt stress. From 1993 to 2023, China, the United States, and India have been major contributors to oat research in both drought and salt-stress fields, and they have maintained high citation counts. This reflects the academic leadership of these three countries in oat research, underlining the impact and quality of their research results.

**Figure 4:**
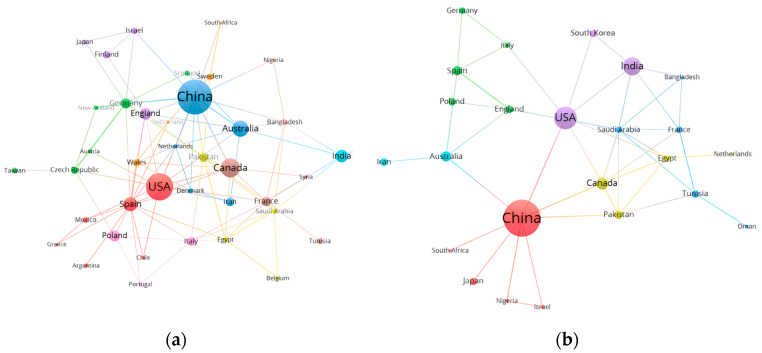
Distribution of oat papers in countries/regions from 1993 to 2023. (**a**) drought stress; (**b**) salt stress. The size of a node indicates the number of papers published in each country. The larger the node, the more papers will be published, and vice versa. The width of the connecting line between nodes indicates the cooperative relationship between countries. The thicker the connecting line, the closer the cooperative relationship, and vice versa.

With the advancement of scientific research, collaboration between different countries has become increasingly common, often yielding greater impact and scientific value. Utilizing VOS cluster analysis, the study examined the network of collaborations among countries with significant involvement in oat research under drought stress (Figure 4a).

The figure presents the results of the cluster analysis for the 42 most connected countries in this research area. It is evident from the figure that China, the United States, Canada, and Australia have the most prominent nodes within the network, reflecting their strong collaborations and high number of publications in the field of drought-stress effects on oat. These connections highlight the intensive cooperation and the central role these countries play in advancing research on drought-stress impacts on oat.

Figure 4b presents a cluster analysis of the 24 countries that collaborate most closely in the field of research on the response of oat to salt stress. The analysis reveals that China, the United States, and India have the most prominent nodes and the greatest connectivity, indicated by the width of the lines between them. This suggests that these three countries have made significant contributions to the study of the effects of salt stress on oat and have established the strongest collaborative relationships within this research domain.

Overall, from 1993 to 2023, China and the United States have emerged as key contributors to oat research in both drought and salt-stress fields, maintaining a consistently high citation record that underscores the impact and quality of their scholarly work. This highlights the importance of international collaboration in advancing scientific understanding and addressing global agricultural challenges.

### 2.3. Publisher’s Citations and Sources of Analysis

The H-index is a valuable metric for assessing the academic impact of journals or individual authors. It reflects the number of publications (H) that have received at least H citations, serving as an indicator of a journal’s or researcher’s academic significance. In the context of oat drought research, Field Crops Research holds the highest H-index of 12, highlighting its influence and importance in the field (Table 5).

In terms of total citations, Proceedings of the National Academy of Sciences of the United States of America leads with 942 citations for papers on drought stress on oat. Following closely are Physiologia Plantarum, with 711 citations, and Field Crops Research, with 480 citations (Table 5). These high citation counts not only underline the quality and impact of the research published in these journals but also illustrate their pivotal role in advancing our understanding of oat responses to drought stress. This emphasis on the H-index and citation totals demonstrates the journals’ central positions in disseminating influential research within the plant science community.

In the realm of salt-stress research on oat, Plant Science leads the field with 1245 citations, demonstrating its significant influence and the high relevance of its published work (Table 6). Following closely are Physiologia Plantarum, with 859 citations, and the Proceedings of the National Academy of Sciences of the United States of America, which has garnered 529 citations. Notably, Physiologia Plantarum stands out with the highest H-index in this area of research, underscoring its pivotal role in disseminating impactful and frequently cited research.

These findings collectively highlight the key focal areas within the response of oat to drought and salt stresses, spanning plant science, agricultural science, environmental science, and ecology. The distribution of publications and the citation trends among the leading journals reflect the significant impact these journals have in advancing the understanding of how oat responds to environmental stresses, therefore shaping the direction and focus of future research in these interconnected fields.

### 2.4. Analysis of the Co-Citation Network

The frequency of citations a publication receives is often reflective of its impact and quality, both nationally and internationally. The analysis curated the top 20 cited papers on the response of oat to drought stress and salt stress, respectively, as recorded in the Web of Science platform (Table 7 and Table 8). These top 20 cited papers on the response of oat to drought stress and salt stress have some similarities in their research areas, which can be roughly summarized as:

(a) Physiological Changes and Responses: Notable contributions include those by Qi and Bai, who provided insights into the physiological and biochemical response mechanisms of oat under stress [15,35]. They explored the effects of drought stress on lipid peroxidation and antioxidant enzymes across various oat varieties. Zhao discussed the physiological responses of oat to key environmental stress factors at different stages [36]. Wang examined the role of stomatal closure and osmotic adjustment in drought adaptation, linking the higher yield of certain oat genotypes to drought tolerance strategies [37]. Additionally, Gao investigated the effect of melatonin on the antioxidant capacity of ruderalis under salt stress and analyzed the antioxidant mechanisms [38].
(b) Yield and Quality: Pandey used oat as a model in field trials to report on scientific advancements regarding oat water use [39]. Sudras examined factors influencing oat yield under stresses [40], while Islam reviewed the impact of drought stress on oat productivity and growth [41]. The physiological mechanisms of drought stress on photosynthesis (Pn) and yield formation were also investigated by Zhao under two different genotypes, where drought stress decreased both oat yield and harvest index at the tassel stage [36]. Additionally, some researchers have analyzed that inoculation with IG3 strain can reduce the effect of salt stress on oat yield and growth under sodium chloride stress [42].
(c) Technology Application: Researchers like Gao and Varghese explored the effects of exogenous melatonin (MT) on the antioxidant capacity of oat during drought and salt stresses, discussing how melatonin alters the plants’ light organs and antioxidant capacity to enhance stress resistance [42,43,44,45]. Sapre and Chang evaluated the inoculation effect of rhizosphere bacteria strain IG3, revealing its potential as a bio-fertilizer in saline-alkali land [21,42]. Song investigated how nitrogen application affects oat photosynthesis under salt stress, finding that increased nitrogen supply maintains photosynthesis and mitigates the adverse effects of stress [46].
(d) Breeding: Studies by Willenborg and Gong focused on the types of oat that exhibit drought resistance and the assessment of oat variants’ biomass using molecular breeding, providing a theoretical foundation for developing high-yielding oats [47,48]. Additionally, Oraby studied third-generation transgenic oat and showed that transgenic R3 plants grew better and were more tolerant to salt stress [49].
(e) Metabolism and Signal Transduction: Researchers like Sanchez, Zhao, and Xu compiled data on metabolic regulation under salt stress, emphasizing the timing, regulation, and integration of these stresses and their impact on stress resistance [50,51,52]. Xu revealed the adaptive response mechanisms of two oat cultivars under salt stress using their metabolomics and transcriptomics approaches. Heald and Wu elucidated the general pathway of stress signal transduction, emphasizing the significance of early induction of signal transduction-related secondary metabolic pathway enrichment in drought-stress management [53,54].

**Table 7:** Literature list of oat drought stress cited in the top 20 from 1993 to 2023.

Ranking	TC	Title	Author/Year	DOI
1	94	Soil Management and Supplemental Irrigation Effects on Potato: I. Soil Properties, Tuber Yield, and Quality	Zhang, M. (1999) [55]	10.1016/S2095-3119(16)61515-0
2	64	Effects of Melatonin on Antioxidant Capacity in Naked Oat Seedlings under Drought Stress	Gao, W. (2018) [43]	10.21930/Rcta.Vol23_Num1_Art:2214
3	63	Oat Germination Characteristics Differ Among Genotypes, Seed Sizes, and Osmotic Potentials	Willenborg (2005) [48]	10.1002/Jsfa.12504
4	55	A Metabolomic Study in Oats (*Avena sativa*) Highlights A Drought Tolerance Mechanism Based upon Salicylate Signaling Pathways and The Modulation of Carbon, Antioxidant and Photo-Oxidative Metabolism	Heald, J. (2015) [50]	10.1590/S0100-83582012000100001
5	48	Fatty Acid Profile Changes During Gradual Soil Water Depletion in Oats Suggests a Role for Jasmonates in Coping with Drought	Sanchez. (2018) [54]	10.3390/Agriculture13020243
6	46	Drought Stress Induced Changes in Lipid Peroxidation and Antioxidant System in Genus Avena	Pandey, H. (2010) [39]	10.1016/0005-2736(91)90195-E
7	38	The Optimum Ridge-Furrow Ratio and Suitable Ridge-Covering Material in Rainwater Harvesting for Oats Production in Semiarid Regions of China	Qi, W. (2015) [56]	10.1016/0005-2736(92)90253-I
8	34	Effects of water-saving superabsorbent polymer on antioxidant enzyme activities and lipid peroxidation in Oat (*Avena sativa* L.) under drought stress	Islam, M. (2011) [41]	10.1002/jsfa.4234
9	30	Early Activation of Plasma Membrane H^+^-Atpase and Its Relation to Drought Adaptation in Two Contrasting Oat (*Avena sativa* L.) Genotypes	Gong, D. (2010) [47]	10.1016/j.envexpbot.2010.02.011
10	28	Evaluation of Drought Tolerance Indices for Selection of Turkish Oat (*Avena saliva* L.) Landraces under Various Environmental Conditions	Akcura, M. (2011) [57]	10.1016/j.tripleo.2009.03.011NA
11	25	Higher Rust Resistance and Similar Yield of Oat Landraces Versus Cultivars Under High Temperature And Drought	Dunn, M. (2017) [58]	10.1007/Bf00267460
12	22	Source-Sink Adjustment: A Mechanistic Understanding of the Timing and Severity of Drought Stress on Photosynthesis and Grain Yields of Two Contrasting Oat (*Avena sativa* L.) Genotypes	Zhao, B. (2021) [36]	10.1007/s00344-020-10093-5
13	22	Oat Phenotypes for Drought Adaptation and Yield Potential	Sadras, V. (2017) [40]	10.1111/1365-2656.13189
14	20	Recently-Released Genotypes of Naked Oat (*Avena nuda* L.) Out-Yield Early Releases under Water-Limited Conditions By Greater Reproductive Allocation and Desiccation Tolerance	Wang, T. (2017) [37]	10.3390/Agriculture11040332
15	19	Salicylic Acid Regulates Polyamine Biosynthesis during Drought Responses in Oat	Yule, K.(2015) [59]	10.1007/S11829-010-9112-5
16	17	Effect of Seed Size and Drought Stress on Germination and Seedling Growth of Naked Oat (*Avena sativa* L.)	Mut, Z. (2010) [60]	10.1016/S1671-2927(09)60153-X
17	16	Influence of Water Stress on Absorption, Translocation and Phytotoxicity of Fenoxaprop-Ethyl and Imazamethabenz-Methyl in Avena Fatua	Xie, H. (1996) [61]	10.1111/j.1365-3180. 1996.tb01802.x
18	15	Targeting Sources of Drought Tolerance Within an *Avena* spp. Collection through Multivariate Approaches	Lin, Y. (2012) [62]	10.1007/S00572-020-00963-X
19	15	Water Use Efficiency and Physiological Responses of Oat under Alternate Partial Root-Zone Irrigation in The Semiarid Areas of Northeast China	Kavroulakis, N. (2012) [63]	10.1016/J.Plaphy.2021.08.029
20	14	Application of Photochemical Parameters and Several Indices Based on Phenotypical Traits to Assess Intraspecific Variation of Oat (*Avena sativa* L.) Tolerance to Drought	Arabia, S. (2017) [64]	10.1007/S12298-021-01023-0

**Table 8:** Literature list of oat salt stress cited in the top 20 from 1993 to 2023.

Ranking	TC	Cited Document	Author/Year	DOI
1	150	Growth, Gas Exchange, Chlorophyll Fluorescence, and Ion Content of Naked Oat in Response to Salinity	Zhao, G. (2007) [23]	10.2135/cropsci2006.06.0371
2	110	Klebsiella Sp Confers Enhanced Tolerance to Salinity and Plant Growth Promotion in Oat Seedlings (*Avena sativa*)	Sapre, S. (2018) [42]	10.1016/j.micres.2017.09.009
3	98	Plant Growth-Promoting Bacteria Facilitate the Growth of Barley and Oats in Salt-Impacted Soil: Implications for Phytoremediation of Saline Soils	Chang, P. (2014) [21]	10.1080/15226514.2013.821447
4	73	Effects of Silicon Nanoparticles on Molecular, Chemical, Structural, and Ultrastructural Characteristics of Oat (*Avena sativa* L.)	Asgari, F. (2018) [65]	10.1016/J.Plaphy.2018.03.021
5	59	Melatonin-Mediated Regulation of Growth and Antioxidant Capacity in Salt-Tolerant Naked Oat under Salt Stress	Gao, W. (2019) [38]	10.3390/ijms20051176
6	48	Salicylic Acid, Hydrogen Peroxide and Calcium-Induced Saline Tolerance Associated with Endogenous Hydrogen Peroxide Homeostasis in Naked Oat Seedlings	Xu, Q. (2018) [66]	10.1007/s10725-007-9247-2
7	40	Transcriptome Analysis of Hexaploid Hulless Oat in Response to Salinity Stress	Wu, B. (2017) [53]	10.1371/journal.pone.0171451
8	32	Melatonin Positively Influences the Photosynthetic Machinery and Antioxidant System of *Avena sativa* during Salinity Stress	Varghese, N. (2019) [44]	10.3390/plants8120610
9	27	Integrative Analysis of Transcriptome and Metabolome Reveal Mechanism of Tolerance to Salt Stress in Oat (*Avena sativa* L.)	Xu, Z. (2021) [51]	10.1016/j.plaphy.2021.01.027
10	26	Barley Hva1 Gene Confers Salt Tolerance in R3 Transgenic Oat	Oraby, H. (2005) [49]	10.2135/cropsci2004-0605
11	26	Nitrogen Application Improved Photosynthetic Productivity, Chlorophyll Fluorescence, Yield and Yield Components of Two Oat Genotypes under Saline Conditions	Song, X. (2019) [46]	10.3390/agronomy9030115
12	25	Influence of Gibberellic Acid and Different Salt Concentrations on Germination Percentage and Physiological Parameters of Oat Cultivars	Chauhan, A. (2019) [67]	10.1016/j.sjbs.2019.04.014
13	25	Physiological and Tmt-Based Proteomic Analysis of Oat Early Seedlings in Response to Alkali Stress	Zhao, Z. (2019) [52]	10.1016/j.jprot.2018.12.018
14	23	Screening Oat Genotypes for Tolerance to Salinity and Alkalinity	Bai, J. (2018) [14]	10.3389/fpls.2018.01302
15	23	Physiological and Biochemical Changes of Cbf3 Transgenic Oat in Response to Salinity Stress	Oraby, H. (2012) [68]	10.1016/j.plantsci.2012.01.003
16	23	Potential Application of Oat for Phytoremediation of Salt Ions in Coastal Saline-Alkali Soil	Han, L. (2012) [16]	10.1016/j.ecoleng.2013.09.034
17	23	Proteomic Response of Oat Leaves to Long-Term Salinity Stress	Bai, J. (2016) [69]	10.1007/s11356-016-8092-0
18	22	Effect of Alkali Stress on Soluble Sugar, Antioxidant Enzymes and Yield of Oat	Bai, J. (2013) [70]	10.1016/S2095-3119(13)60556-0
19	19	Effects Of Saline and Alkaline Stresses on Growth and Physiological Changes in Oat (*Avena sativa* L.) Seedlings	Gao, Z. (2014) [71]	10.15835/nbha4229441
20	19	Use of Mixed Solid Waste as A Soil Amendment for Saline-Sodic Soil Remediation and Oat Seedling Growth Improvement	Fan, Y. (2019) [72]	10.1007/s11356-016-7360-3

Overall, the response of oat to drought and salt stresses is multifaceted, encompassing physiological changes, yield and quality, cultivation, and breeding, as well as metabolism and signaling. In the field of drought-stress research, the focus is primarily on physiological changes, yield and quality, and the breeding of oat. Meanwhile, in the field of salt-stress research, the emphasis is mainly on physiology, metabolism, ionic homeostasis, and breeding. These studies have explored the effects of drought and salt stress on oat from multiple perspectives and have provided far-reaching insights into the relationship between oat growth and stress, as well as the mechanisms for coping with stress.

### 2.5. Analysis of the Co-Occurrence Network

**Figure 5:**
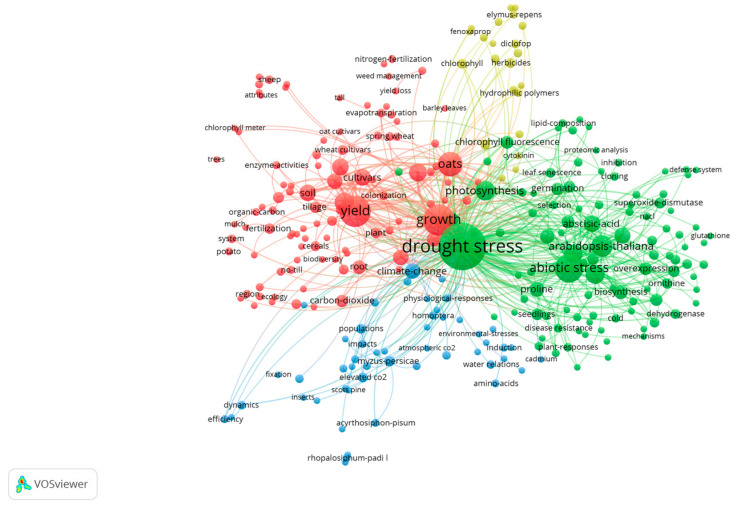
Co-occurrence analysis of key words drought stress in oat (f ≥ 2). Node size represents the frequency of keywords, and the larger the node, the higher the frequency, and vice versa. The line connecting the nodes indicates that two keywords plus appear in the same cited document, and the thicker the line, the higher the frequency of occurrence, and vice versa.

Keywords not only reveal the direction of research but also map the evolution of research hotspots. In exploring the research area of the effects of drought stress on oat, we conducted a cluster analysis of keywords with a frequency of at least two occurrences, as shown in Figure 5. This analysis divided the research themes into four main clusters, each highlighting a unique academic focus:

**Red Cluster (266 items):** Concentrating on oat, growth, yield, quality, breeding, and variation, this cluster explores the ramifications of drought stress on oat growth, yield, and quality. The focal point here is on improving oat production, quality, and variety through targeted breeding efforts and stress management strategies.

**Green Cluster (102 phrases):** Encompassing terms such as photosynthesis, drought, *Arabidopsis thaliana*, and abiotic stress, the research within this cluster aims to understand how photosynthesis adapts under drought stress. It also delves into gene expression, as well as the physiological and biochemical responses of *Arabidopsis thaliana* to drought, offering insights into broader plant stress resistance mechanisms.

**Blue Cluster (51 words):** Covering terms like climate change, environmental stress, carbon dioxide, and physiological response, this cluster investigates the physiological responses of plants to environmental stressors and climate change, focusing on adaptive strategies in the face of increasing environmental challenges.

**Yellow Cluster (22 terms):** The cluster focuses on the effects of drought stress on seed production management and plant physiological and biochemical responses, with particular attention to the key areas of ascorbate peroxidase, seed germination, and chlorophyll. The study emphasizes the importance of seed germination and early plant development under stress conditions and the physiological and biochemical responses of plants to such adversities. Additionally, the research addresses the role of oat in medical therapeutic areas, particularly keywords such as fenoxaprop and hydrophilic polymers. These studies highlight the potential value of oat in drug development. In the area of cultivation and management, keywords such as elymus-repens, diclofop, and herbicides are focused on the cultivation techniques and management methods of oat. These studies aim to improve the yield and quality of oat and optimize cultivation practices.

**Figure 6:**
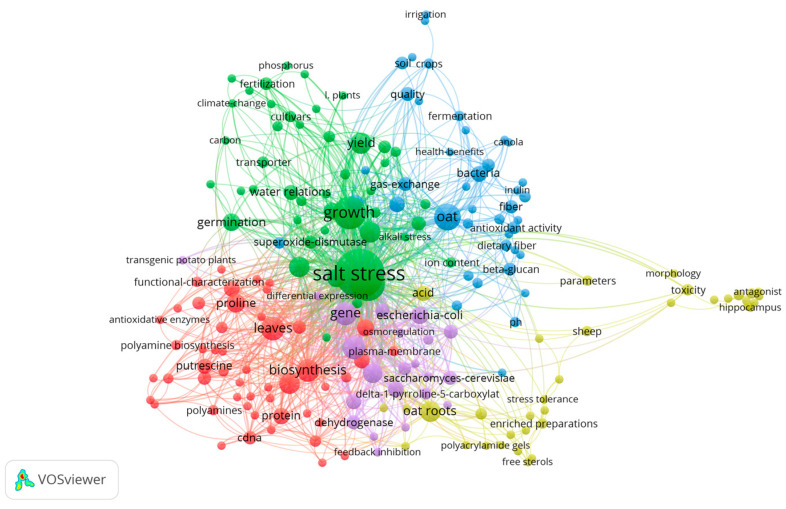
Co-occurrence analysis of key words salt stress in oat (f ≥ 2). Node size represents the frequency of keywords, and the larger the node, the higher the frequency, and vice versa. The line connecting the nodes indicates that two keywords plus appear in the same cited document, and the thicker the line, the higher the frequency of occurrence, and vice versa.

To delve deeper into the research area of the effects of salt stress on oat, we conducted a cluster analysis of keywords that appeared at least twice in frequency, and the results are shown in Figure 6. This analysis revealed five major clusters of research themes, each representing a unique academic research focus:

**Red Cluster (50 terms):** This segment focuses on abscisic acid, proline, protein, metabolism, biosynthesis, and leaves, with a primary emphasis on metabolic and biosynthetic pathways and their alterations in response to salt stress, aiming to understand nutrient production and metabolic changes, shedding light on how these processes adapt to enhance plant resilience under saline conditions.

**Green Cluster (45 keywords):** Pertaining to abiotic stressors such as salinity, yield, germination, growth, and photosynthesis, this cluster’s research addresses the impact of such stressors on germination, growth, yield, and quality, contributing valuable insights into stress adaptation and management, helping to formulate strategies that can mitigate the adverse effects of salt stress on crop performance.

**Blue Cluster (41 terms):** Focusing on oat, gas exchange, and quality, research within this cluster explores the implications of salt stress on photosynthesis and oat quality. It underscores the interconnectedness of physiological processes and product quality under stress, highlighting the critical role of maintaining photosynthetic efficiency and gas exchange in sustaining crop health and output.

Yellow Cluster (35 keywords): The main research directions of this cluster focus on three high-frequency keywords: oat root system, toxic effects, and soil acidity. y explore how elevated soil acidity affects ion accumulation in oat roots and investigate the mechanisms by which the root system responds to increased toxicity and maintains its function under adverse conditions. Additionally, research keywords include polyacrylamide gel, free sterols, and morphology. Polyacrylamide gel electrophoresis has been used to isolate and analyze proteins extracted from oat leaves or roots, providing insights into protein expression patterns in oat under specific environmental stresses such as salt stress. Salt stress damages plant cell membranes by increasing extracellular fluid osmotic pressure, leading to cellular dehydration. Free sterols are essential for maintaining cell membrane integrity and function. Under salt-stress conditions, the morphological characteristics of oat leaves and roots are adversely affected, which is closely related to physiological and biochemical changes in oat.

Purple Cluster (22 terms): Including genes, cold stress, and Arabidopsis thaliana, this cluster leverages Arabidopsis thaliana as a model organism to investigate the genetic underpinnings of stress resistance, aiming to enhance yield under adverse climate conditions by applying insights gained from genetic studies on stress tolerance, potentially transferring successful strategies to oat cultivation.

After analyzing the clustering of keywords, we found that the red clustering involving drought stress and the green clustering of salt-stress studies focused on the growth, yield, and quality of oat under various stress conditions. Meanwhile, the green clustering of drought stress and the blue clustering of salt stress focused on the changes in photosynthesis when oat are exposed to abiotic stresses and the effects of environmental stresses on oat’s physiological processes and product quality. In addition, the green keyword clustering for drought stress and the purple clustering for salt stress aims to enhance the yield of oat under adverse conditions by investigating the genetic basis of plant resilience using *Arabidopsis thaliana* as a model organism.

### 2.6. Keywords Centrality and Outbreak Analysis

The importance of keywords can be assessed by their centrality, where high-frequency keywords not only reveal hot topics in the field of research but also demonstrate their strong connections with other keywords. In the research on the effects of drought stress on oat, Table 9 shows that among the top 15 keywords, “drought stress” and “oats” have the highest centrality, indicating a strong connection between these two subject terms and other keywords. Keywords such as “leaf”, “yield”, and “quality” emphasize the effects of drought stress on oat growth and biomass, making it a hot research topic. Additionally, the keywords like “abscisic acid”, “climate change”, “photosynthesis”, and “oxidative stress” reveal the significant impacts of extreme climate change (e.g., severe drought and temperature extremes) on the physiology and biochemistry of oat, which is another research focus. Meanwhile, keywords such as “Arabidopsis thaliana”, “variety”, and “gene” highlight the importance of oat breeding research under drought stress.

In the research on the effect of salt stress on oat, keywords like “salt stress”, “oat”, “abscisic acid”, “growth”, and “arginine decarboxylase” have high centrality and frequency, indicating a close relationship among these terms. The keywords “biosynthesis”, “oxidative stress”, “proline”, and “metabolism” focus primarily on the physiological and biochemical effects on oat under salt-stress conditions.

**Figure 7:**
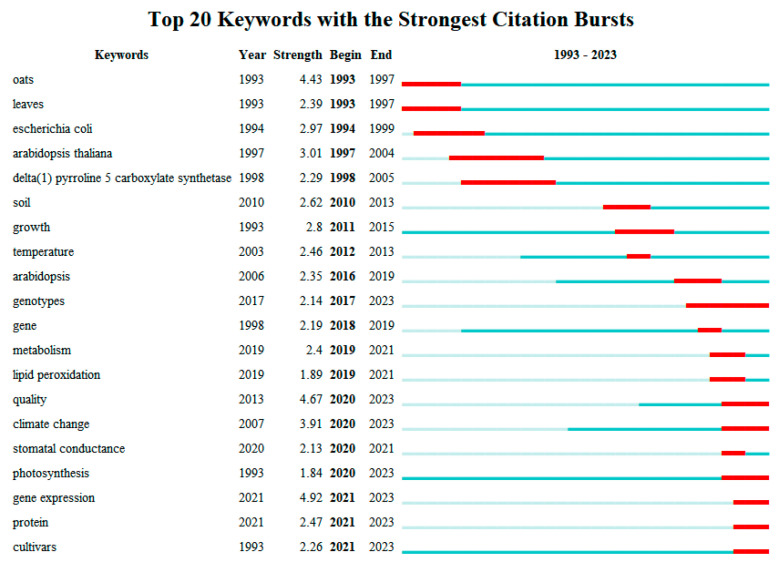
Top 20 keywords with the strongest citation bursts drought stress on oat. The blue and red indicate the time axis (1993–2023) and burst period in the emergence burst column, respectively.

The exploration of keywords’ centrality and trends offers a detailed snapshot of the dynamic research landscape pertaining to the oat response to drought and salt stress. In the field of drought-stress research, the chronological progression of research focus reveals notable shifts (Figure 7):

**1993–1998:** Research initially homed in on the impact of drought stress on oat roots and leaves, including alterations in enzyme activities and proline content, highlighting these aspects as primary research hotspots.

**1999–2019:** The focus expanded to encompass growth and yield, physiological and biochemical modifications, and the development of transgenic oat under drought stress. This era also saw transgenic studies using Arabidopsis as a medium for exploring oat responses to salt stress.

**2020–2023:** The latest research trends emphasize growth and gene expression in oat subjected to drought stress, underscoring a heightened interest in not only the survival mechanisms of oat under stress conditions but also in how these stressors influence the crop’s nutritional quality and overall value.

**Figure 8:**
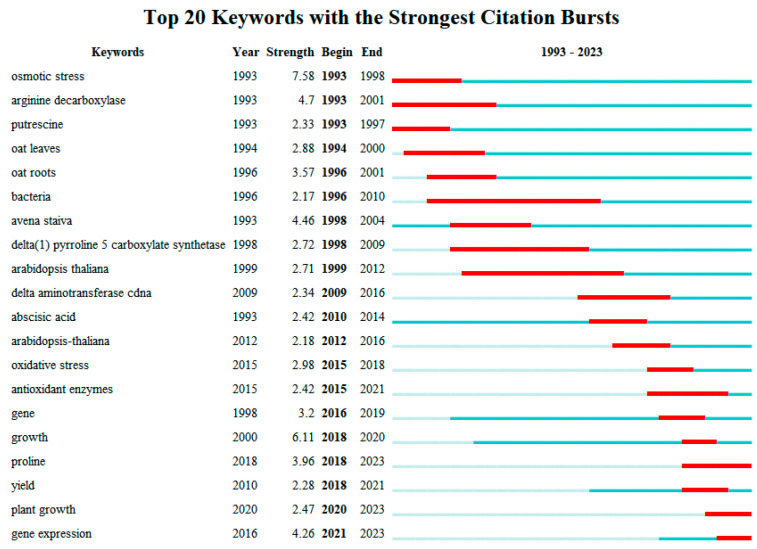
Top 20 keywords with the strongest citation bursts salt stress on oat. The blue and red indicate the time axis (1993–2023) and burst period in the emergence burst column, respectively.

In the field of salt-stress research, the chronological progression of research focus reveals notable shifts (Figure 8):

**1993–1998:** Research initially homed in on the impact of salt stress on oat roots and leaves, including alterations in enzyme activities and proline content, highlighting these aspects as primary research hotspots.

**1999–2019:** The focus expanded to encompass growth and yield, physiological and biochemical modifications, and the development of transgenic oat under salt stress. This era also saw transgenic studies using Arabidopsis as a medium for exploring oat responses to salt stress.

**2020–2023:** The latest research trends emphasize growth and gene expression in oat subjected to salt stress, underscoring a heightened interest in not only the survival mechanisms of oat under stress conditions but also in how these stressors influence the crop’s nutritional quality and overall value.

By analyzing the evolution of the keywords, as time progressed, researchers not only focused on the effects of drought and salt stress on the external morphological structure of oat but also delved into the effects of these two adversity conditions on the growth, yield, and physiological characteristics of oat. Under drought conditions, studies have particularly examined oat growth, yield formation, and genetic improvement, whereas under salt stress, the emphasis has been on oat growth performance, yield, and the development of salt-tolerant varieties. Regardless of whether it is under drought or salt stress, the growth, genetic breeding, and stress tolerance mechanisms of oat have consistently been the focus of researchers. In addition, the level of research shifted from intuitive morphological traits to genetic and molecular mechanisms, ultimately focusing on how environmental stress affects the nutritional value of oat and its impact on the overall value of the crop.

## 3. Discussion

The gradual escalation of global warming and soil salinization poses subtle yet significant challenges, leading to abiotic stressors such as drought, induced by heightened temperatures, and salt stress, resulting from salinized soils, affecting diverse regions worldwide. These environmental adversities exert profound detrimental impacts on oat crops, manifesting in stunting, retarded growth, diminished yields, and, in extreme scenarios, plant mortality [7,73]. The imperative to investigate the effects of drought and salt stress on oat is underscored by the necessity to devise adaptive strategies that can mitigate the growing prevalence of these conditions.

### 3.1. Yield and Quality

A pivotal challenge within plant science is the dichotomy between achieving high yield and preserving stress tolerance. This conundrum has been an enduring goal within the discipline. Moisture stress is an important factor affecting the yield of oat, and the extent of its effect is related to the duration and intensity of the stress [47,74]. It was found that 15 days of water deficit stress resulted in yield reduction in both naked and hulled white oat and that drought affected yield formation by reducing the number of spikelets in oat genotypes [75]. Meanwhile, the recently released (RR) genotype oat outperformed earlier released (ER) genotypes in terms of yield, harvest index (HI), and grain water use efficiency. Aboveground biomass and HI significantly influenced grain yield, while leaf dry weight was negatively correlated with grain yield [37]. Yield differences among genotypes were not completely consistent across moisture conditions, with RR genotypes being more adapted to drought under moderate soil content conditions but not under low soil water content conditions. Higher yielding RR genotypes were not directly related to aboveground biomass but to higher HI, better grain water use efficiency, and larger seed size. This is consistent with studies on other cereal crops where grain yield has increased over time mainly due to increased HI rather than aboveground biomass [76].

Soil salinity also adversely affects oat growth and yield, with salt stress leading to a decrease in plant biomass and grain yield at harvest associated with photosynthetic productivity, chlorophyll fluorescence, carbohydrate metabolism, and assimilate translocation [77]. Elevated soil salt concentrations disrupt normal plant physiological functions by increasing cellular Na+ levels, which interfere with the uptake of essential minerals such as Mg^2+^ [5,14,21]. Such conditions negatively impact photosynthesis in oat by affecting chlorophyll synthesis and concentration [19,78,79]. Given chlorophyll’s pivotal role in photosynthesis, a decrease in its concentration directly diminishes photosynthetic efficiency, consequently influencing growth rates, biomass accumulation, and overall oat yield. However, plant response to salinity changes varies with salinity treatments and methods of saline use during the growth phase [80]. Under low and moderate salinity conditions, fertilization can mitigate the inhibitory effects of salt stress on oat yield and growth and enhance nitrogen uptake [81]. Furthermore, research by Sapre, S highlights that salt tolerance in oat can be augmented, and their yield increased through the application of the salt-tolerant rhizosphere strain IG3, derived from the rhizosphere of wheat plants [42,82]. Therefore, the effects of drought and salt stresses on oat yield can be mitigated by selecting appropriate genotypes and rational fertilization.

Overall, the effects of drought and salt stress on oat yield were mainly reflected through the following key factors: spikelet number, genotype, water use efficiency, photosynthesis, and metabolite production. These stress conditions lead to a decrease in the performance of these factors, which in turn reduces oat yield and biomass.

### 3.2. Molecular Response

#### 3.2.1. Metabolomics and Transcriptomics

While much of the research continues to focus on improving crop yield and stress tolerance in oat, genomics research is emerging as a promising avenue to address drought and salt stress effectively in certain locales. In response to adversities such as high salinity and drought, plants protect themselves by adjusting the composition of metabolites and the production of secondary metabolites. Using transcriptomic and metabolomic approaches, recent studies have delved into how oat responds to abiotic adversity through complex biological pathways, revealing that alterations in gene expression are not always directly related to changes in metabolites, as metabolic regulation involves the interaction of numerous genes [82,83].

Hydrogen peroxide acts as a key signaling molecule that is not only involved in plant physiological processes but also extensively involved in cross-resistance responses [84]. Studies have shown that hydrogen peroxide plays a key role as a second messenger in plant resistance signaling mediated by brassinosteroids (BRs) and abscisic acid (ABA) [85]. Researchers Xia et al. proposed that BRs might trigger the MAPK cascade reaction through the hydrogen peroxide pathway to regulate the expression of antioxidant enzyme genes and increase the activities of antioxidant enzymes in cucumber, therefore enhancing its stress resistance [85].

Under drought conditions, the leaves of oat produce large amounts of hydrogen peroxide. Hydrogen peroxide, as a second messenger, can activate the MAPK cascade reaction and up-regulate the expression of antioxidant-related transcription factor genes, contributing to enhanced tolerance in oat seedlings [86,87]. Phosphorylated MAPK acts as a bridge in this process, enhancing the expression of transcription factors such as NAC, WRKY1, DREB2, and MYB in oat, which in turn activates downstream genes and improves the plant’s drought tolerance. Melatonin (MT) has mechanistic similarities with BRs. In oat, Asmap1 and Aspk11 are two MAPKs induced under drought conditions with or without the presence of MT [88]. The relative expression levels of Asmap1 and Aspk11 were significantly up-regulated under drought stress and were involved in regulating the MAPK cascade through the hydrogen peroxide pathway. The relative expression levels of these two MAPKs were found to be higher in drought+MT-treated plants, suggesting that MT might induce MAPK cascade responses through the hydrogen peroxide pathway to further enhance drought tolerance in oat seedlings [42].

When oat is exposed to salt stress, the molecular response is based on the perception and signaling transduction mechanisms of the stress. This process typically begins with the recognition of the stress signal, followed by the activation of a series of secondary messengers, such as changes in intracellular calcium concentration, hormone synthesis, and protein phosphorylation cascades, ultimately directly affecting the activity of transcription factors and controlling the expression of genes that regulate specific stress responses. To adapt to this stress, oat has established signaling and pathway mechanisms to adjust ion homeostasis [89]. Under salt stress, oat regulates metabolites and related pathways through changes in the transcriptome to enhance its salt tolerance [90]. Salt stress inhibits oat growth, especially in saline soils, and leads to increased levels of reactive oxygen species (e.g., hydrogen peroxide), which in turn activate calcium channels and promote the flow of intra- and extracellular calcium ions [91,92]. Increased cytoplasmic calcium ion levels can either activate NADPH oxidase to produce hydrogen peroxide or reduce hydrogen peroxide levels through Ca^2+^/CaM-mediated enhancement of catalase enzyme (CAT) activity, suggesting that cytoplasmic calcium ions have a dual role in regulating redox homeostasis, which in turn affects oat’s response to environmental stimuli [93]. In addition, salt stress leads to elevated levels of sodium ions in plants, which triggers significant toxic effects [94]. Accumulation of sodium ions inhibits the uptake of essential ions, such as calcium and potassium, resulting in ion homeostasis imbalance, which may be the main reason for growth limitation, biomass reduction, and increased osmotic and oxidative stress in plants [4]. In summary, oat exhibits a range of adaptive responses when faced with drought and salt stress. Through transcriptomics and metabolomics studies, we found that oat mitigates the damage caused by stress by regulating gene expression, changing the direction of ion flow, and adjusting hormone signaling. In addition, we have gained a deeper understanding of the mechanisms and pathways by which oat resists drought and salt stress, which provides a scientific basis for preventing abiotic stresses from adversely affecting oat growth in the future.

#### 3.2.2. Proteomics

AQPs are members of the major intrinsic proteins (MIP) superfamily and are crucial for regulating plant growth and development [95] due to their role in about 70% of water transport across membranes. Several genomic analyses of AQPs in higher plants have shown that these proteins play an essential role in responding to various abiotic stresses such as salinity, drought, and extreme temperatures, which can impair water uptake in plants [96]. Research into the expression patterns of wheat and oat AQP genes under conditions like extreme temperatures, salt, and drought stress suggests that most AsAQP genes may be involved in coping with these stresses. Drought stress has been found to suppress the expression of AsAQP family genes, indicating a potential negative regulatory role in molecular pathways that enhance drought acclimatization in both wheat and oat [97]. Furthermore, in a related study, a novel β-amylase enzyme named AsBAMY was isolated and characterized from oat-seed extract. AsBAMY showed high homology with known plant β-amylase family members. Bioinformatics analysis predicted the three-dimensional structure of AsBAMY, revealing its typical (β/α) 8-barrel core structure with the catalytic active site located at one end of the barrel. This suggests that AsBAMY functions as an exo-cleavage hydrolase. The study of the flexible loop regions of AsBAMY indicated that conformational changes in these regions are critical for enzyme activity, aligning with previous findings on β-amylase. These results further emphasize the importance of studying the AsAQP gene for barley germplasm resources, and molecular-assisted breeding and understanding of oat-seed amylase results provide valuable information for further research on the genome [98].

Overall, the ability of plants to absorb water is compromised under drought and salt stress due to suppressed expression of aquaporin genes. Understanding the protein functions in oat proteomics under these stress conditions lays a theoretical foundation for improving oat germplasm resources and molecular breeding strategies.

#### 3.2.3. Genomics

The study of genomics is an intuitive representation of the molecular responses of plants to abiotic stress. Through genomic research, scientists can uncover the molecular response mechanisms of plants under environmental pressures such as drought, salt, and low temperatures. Genomic studies of the structure of related genes in the oat gene family can lead to a more comprehensive understanding of the molecular response mechanisms of oat in drought and salt-stress environments. The water channel protein gene (AsAQP) is an important molecule in the oat (*Avena sativa*) genome for molecular response. Real-time quantitative PCR has verified the expression patterns of these AsAQP genes in various oat tissues under different abiotic stress conditions, enhancing our understanding of their functional roles [95]. Furthermore, a comparison with other species revealed that the number of AsAQP family members in the wheat (*Triticum aestivum*) genome surpasses those in Arabidopsis (*Arabidopsis thaliana*), rice (*Oryza sativa*), and maize (*Zea mays*). This diversity and the apparent replication events during plant evolution suggest functional divergence among AQP genes [99], which may have adapted different biological functions.

Additionally, a genome-wide study by Mouna Ghorbel on the catalase (CAT) gene family in oat explored the potential roles of these genes under salt stress. Using bioinformatics approaches, the study predicted the structure, secondary and tertiary protein structures, physicochemical properties, phylogenetic relationships, and expression profiles of AvCAT genes under various developmental and biological conditions [100]. The findings suggest that oat CAT genes are differentiated into groups based on their function in photosynthetic, vascular, and reproductive processes, with all identified AvCATs contributing to intracellular oxidant detoxification and hydrogen peroxide degradation.

Moreover, genomic sequencing-based genotyping methods were utilized to analyze 4657 cultivated oat materials. Techniques such as linkage analysis, population genomics, genome-wide association studies, and genomic selection were performed using data from the oat consensus map. The study highlighted the advantages of haplotype markers based on the tag level in linkage analysis, high-resolution genomic analysis, and genomic selection, providing a high-density, highly informative marker system for oat genomic research and molecular-assisted breeding [101]. These comprehensive genomic analyses offer crucial insights into oat adaptation to environmental stresses and potential strategies for crop improvement.

### 3.3. Physiological and Biochemical Response

#### 3.3.1. Antioxidant and Lipid Peroxidation

Under drought and salt stress, reactive substances (e.g., reactive oxygen species, hydrogen peroxide, superoxide anion, etc.) and antioxidant enzymes (e.g., superoxide dismutase SOD, peroxidase POD, catalase CAT, etc.) undergo corresponding changes in response to drought and salt stress. Plant lipids are crucial in responding to environmental stresses, as they are integral to maintaining the structure and function of cell membranes. Studies have shown that in arid environments, the content of leaf polar lipids decreases, which aligns with previous observations of heightened lipolytic activity [62]. This reduction in lipid content can disrupt cell membrane integrity and functionality [102], affecting lipid-protein interactions, enzyme activities, and transport mechanisms essential for cellular processes such as division and bioproduction [103].

In response to drought, oat varieties show varying degrees of lipid and fatty acid composition changes, with drought-susceptible varieties experiencing a significant decrease in total lipid content. This reduction leads to membrane damage and inhibits lipid biosynthesis [104], contributing to the senescence (aging) process of leaves [105]. Moreover, drought stress enhances peroxidase activity and lipid peroxidation, increasing cell membrane permeability, particularly in varieties that exhibit a higher degree of water stress. This makes them more sensitive to drought, yet paradoxically, it may also confer some level of drought tolerance.

Superoxide dismutase (SOD) plays a critical role as a primary defense against reactive oxygen species (ROS). Under drought conditions, a reduction in SOD activity may indicate either a decreased synthesis or enhanced degradation of the enzyme, potentially serving as a protective mechanism against oxidative damage by reducing the accumulation of harmful species like hydrogen peroxide and oxygen [85].

Additionally, oxidative stress is exacerbated by the increased levels of ROS and other reactive metabolites under salt stress, leading to further damage or destruction of cell membranes [106]. However, studies have demonstrated that antioxidant enzyme activities, including those of SOD, peroxidase (POD), and catalase (CAT), are elevated in oat treated with melatonin (MT) in saline conditions [42]. This enhancement in enzyme activity helps mitigate the effects of salt stress by reducing the accumulation of free radicals and decreasing the production of malondialdehyde [107], a byproduct of membrane lipid peroxidation.

In summary, both drought and salt stresses provoke significant changes in enzyme activities related to oxidative stress management in oat. These changes contribute to the regulation of ROS production, which can intensify lipid peroxidation processes and affect the physiological and biochemical responses of the plants. While these stresses lead to damage and increased sensitivity in plant cells, they may also paradoxically enhance the plants’ resilience to further stress. This dual response underscores the complex nature of plant adaptation to environmental challenges.

#### 3.3.2. Photosynthesis

Photosynthesis is a fundamental physiological process in plants which may be affected under drought and salt stresses conditions. In addition, there are many factors affecting photosynthesis, most notably chlorophyll and proline.

Under salt-stress conditions, plants experience deficiencies in water, minerals, and energy supply, which directly affect their key physiological functions, particularly interfering with photosynthesis. As photosynthesis is the fundamental process for plants to acquire materials and energy, a decrease in its efficiency naturally has a detrimental effect on plant growth and biomass production. Salt stress compromises the integrity of cell membranes, leading to a decrease in chlorophyll content and a reduction in photosynthetic efficiency. Chlorophyll a and chlorophyll b, as the core components of chlorophyll, are responsible for absorbing light of different wavelengths and are crucial for regulating photosynthesis. However, under salt stress, the levels of both chlorophyll a and b decrease, which not only limits the rate of photosynthesis but also reduces the plant’s biomass [108].

The changes in proline and soluble sugar content are important indicators for assessing plant salt tolerance and are key factors in determining photosynthetic activity [54]. In response to salt stress, plants adjust their proline and soluble sugar content. Since salt concentration disrupts the pathways of synthesis and metabolism, these salt-tolerance indicators usually decline, therefore affecting the rate of photosynthesis. However, studies have shown that methods such as inoculating with plant growth-promoting rhizobacteria (PGPR) and treating with melatonin (MT) can increase the content of soluble sugars in plants, thus mitigating the effects of salt stress [109]. Additionally, these treatments can also elevate protein levels, indicating that plants may possess the ability to counteract the oxidative damage caused by reactive oxygen species under salt stress [110,111].

Drought stress damages chloroplasts and inhibits chlorophyll synthesis, leading to a decrease in chlorophyll and reduced photosynthesis [112]. A decrease in chlorophyll concentration in plants may be a stomatal limiting factor, and chlorophyll is less affected by drought in drought-tolerant plants [113]. Studies on legumes, chickpea, and wheat showed that drought stress reduced chlorophyll a and b content [114]. The chlorophyll content of oat varieties decreased after drought stress, which may be a result of damage to chloroplasts and inhibition of chlorophyll synthesis, and drought-tolerant varieties had higher chlorophyll content than drought-sensitive varieties. In addition, the term “temperature” often appears in conjunction with drought-stress research, highlighted as one of the key factors that impact drought-stress. For example, extreme temperatures exacerbate drought and temperature-accelerated drought fluctuations significantly affect plant physiological and biochemical processes, such as photosynthesis, respiration, and water use efficiency. Drought due to increased temperatures closes the stomata, which is a mechanism by which plants regulate the balance of water within their bodies. Second, after drought stress is exacerbated by increased temperatures due to climate warming, the rate of water loss through transpiration from the plant’s stomata increases. To minimize water loss, plants close their stomata. When stomata are closed, the supply of carbon dioxide (CO_2_) inside the plant’s leaves decreases, which directly affects the rate of photosynthesis [77,115,116,117].

These studies indicate that drought and salt stress reduce the contents of chlorophyll, proline, soluble sugar, and protein in oat, which in turn leads to a decrease in leaf water potential and stomatal closure, ultimately inhibiting the photosynthesis of oat. These changes may affect the growth, yield, and quality of oat. However, as the content decreases, oat shows a certain degree of resistance to adverse environmental conditions. This resistance trait provides a potential basis for the screening and breeding of oat varieties adapted to stressful environments.

#### 3.3.3. Plant Hormones

Phytohormones (including plant endogenous and exogenous hormones) play key roles in all stages of plant growth and development, including seed germination, root development, stem extension, flowering, and fruit ripening. In addition, phytohormones play an important role in plant response to environmental stresses such as drought, salt damage, cold, and other adversities.

**(a) exogenous hormones.** The application of exogenous hormones is a well-established method to boost crop resilience to environmental stresses and enhance overall productivity. Studies have shown that exogenous salicylic acid (SA) has been shown to increase salt-stress tolerance in oat seedlings by maintaining photosynthetic pigment levels, enhancing antioxidant defenses, and improving osmotic regulation. Similar benefits have been observed with acetazolamide in corn and acetate in strawberries, both enhancing root growth and nutrient absorption [118], thus improving plant vitality under stress conditions. Similarly, in strawberries, acetate treatment has effectively increased fresh weight and dry weight by promoting the supply of water and nutrients [119]. These findings highlight the potential of acetate-like compounds in improving crop stress resistance, providing a new strategy for crop improvement.

Melatonin (MT), as a naturally occurring active substance, has an exogenous application that promotes the germination of oat seeds under saline stress, and its effect is affected by the application concentration and application time, showing the phenomenon of promotion at low concentration and inhibition at high concentration. This is consistent with the results of Xiao et al. in cotton study [120]. This may be due to the strong reducing ability of MT in plants, which can scavenge excessive reactive oxygen radicals (ROS) in the body and maintain the metabolic balance of intracellular ROS. MT can reduce the effects of salt stress on oat seeds by decreasing hydrogen peroxide content and increasing catalase activity and osmoregulatory substances. However, after exogenous application of high concentration of MT, the plant absorbed more MT from the external environment, and the excess MT in the body may lead to oxidative denaturation of plant proteins [121], which resulted in the accumulation of ROS species in the body and inhibited the germination and growth of oat seeds. GA has the same mechanism of action. Gibberellic acid (GA) is one of the plant growth regulators that improve salt tolerance and reduce the effects of salt stress on plants. Salt concentration significantly reduced stem and root length in oat varieties. Experimental studies have shown that gibberellic acid can reduce the effect of salt stress on oat-seed germination under salt stress, and gibberellic acid significantly promotes branch and root growth in different oat varieties [67].

Additionally, drought stress inhibited the growth of oat seedlings, while MT inhibited drought-stress-induced oat seedling growth. Similarly, seedling growth promotion was observed in soybean [122] and bermudagrass [123] under drought-stress conditions but with different MT pretreatment concentrations. Therefore, the effect of MT on plant growth under drought stress may be related to the different sensitivities of different plants to MT. Sometimes, the exogenous application of betaine can effectively regulate some key physiological and biochemical attributes under plant stress conditions and alleviate the adverse effects of water stress. Exogenous betaine can effectively up-regulate some anatomical features that are directly or indirectly involved in water stress tolerance in oat plants to make plants resistant to harsh environmental conditions [124].

**(b) plant-derived hormone.** Plant endogenous hormones respond to stress through different physiological responses. For example, stomatal closure can be induced by various factors, with the production of abscisic acid (ABA) being a pivotal one, especially under drought conditions. Following drought perception, the ABA response can lead to an early reduction in stomatal conductance, impeding assimilation and root growth, which exacerbates and accelerates the vicious cycle of drought symptoms [125]. ABA enhances a plant’s drought tolerance, and its early accumulation in roots and leaves is a significant response. ABA is also linked to root growth, as demonstrated by the increased root-to-shoot ratio in artificially water-stressed corn seedlings due to ABA buildup [126]. Under conditions of low water potential, ABA accumulation is necessary to sustain root elongation in corn; however, excessive ABA does not further promote elongation and might even slightly inhibit it. Sustaining oat root growth is a critical factor affecting oat’s drought tolerance. Meanwhile, Salicylic acid (SA) participates in a variety of plant processes, including the regulation of plant development and growth, flowering, and maturation, as well as responses to abiotic stresses. Consequently, SA mediates the response to salinity, low temperatures, and drought. SA modulates the levels of polyamines by affecting the expression of its primary biosynthetic genes, therefore alleviating drought symptoms [59].

In conclusion, both exogenous and endogenous plant hormones significantly influence oat seedling growth under stress. They contribute to stress mitigation by enhancing chlorophyll, proline levels, and other vital substances, which in turn improve photosynthetic efficiency and promote seedling growth and biomass accumulation. This hormonal intervention provides a valuable strategy for enhancing crop resilience in adverse environmental conditions.

### 3.4. Cultivation and Breeding

Drought stands out as a critical stressor diminishing plant productivity. Variability in plant response to drought is influenced by factors such as the drought’s severity, the specific crop species, genetic backgrounds, concurrent stresses, and the developmental stage of the plant. Recent studies have explored the genotypic differences in response to soil drought using double haploid (DH) lines derived from pollinated F1 generations of oat (*Avena sativa* L.) and maize [64]. The introduction of maize chromosomes into oat (termed OMA) typically induces both morphological and physiological changes. These include alterations in photosystem II activity and chlorophyll production, which are regulated through interactions between the maize chromosome and the oat genome [127].

Furthermore, β-1,3; 1,4-glucan, a molecule pivotal in environmental response among gramineous plants and a crucial functional component in oat seeds, has been a focus of genetic engineering efforts. For instance, the introduction of the PeNAC1 gene from poplar into hexaploidy cultivated oat resulted in transgenic lines exhibiting significantly enhanced germination rates, survival rates, and chlorophyll content under salt stress compared to non-transgenic controls. This suggests that PeNAC1 improves salt tolerance by maintaining chlorophyll synthesis stability during the nutrient growth stage, thus mitigating yield losses [128]. The expression of PeNAC1 significantly enhanced the germination rate, survival rate, and chlorophyll content of transgenic plants under salt-stress conditions compared to the non-transgenic control. The chlorophyll content of transgenic oat was reduced less and higher than that of non-transgenic oat under salt stress, resulting in reduced yield loss. This suggests that PeNAC1 effectively improves the salt tolerance of transgenic oat during the nutrient growth stage by maintaining the stability of chlorophyll synthesis [64]. These findings provide a theoretical basis for breeding high-yielding and resistant oat varieties.

The study also assessed physiological indicators related to salt stress in transgenic oat during the growth cycle. Seed germination and seedling survival are key indicators of salt-stress resistance in crops. It was shown that transgenic oat exhibited stronger salt tolerance at the seed germination stage, as demonstrated in studies on wheat [129] and oat [14]. During the seedling growth stage, a strong root system is essential for adapting to adverse environments and absorbing nutrients and water to maintain normal plant growth. The root-crown ratio of transgenic oat at the seedling stage was significantly higher than that of non-transgenic varieties, suggesting that salt stress has less negative impact on the root development of transgenic oat.

In summary, the breeding research in oat aims to develop varieties with improved drought and salt tolerance through techniques like genetic engineering and molecular breeding. These approaches facilitate the breeding of novel oat varieties and enhance the performance of existing ones through meticulous selection processes, therefore establishing a robust foundation for the future development of resistant oat varieties. This ongoing research is crucial for ensuring the sustainability and productivity of oat in the face of evolving environmental challenges.

## 4. Materials and Methods

For a comprehensive bibliometric analysis, a robust database collection focusing on the impacts of drought and salt stresses on oat is indispensable. To construct a bibliographic database on oat responses to drought and salt stresses, we utilized the Web of Science Core Collections. Our literature search, employing the terms “drought stress” AND “salt stress,” spanned from 1 January 1993 to 20 November 2023. We conducted this search across all fields, applying Boolean operators to refine our search criteria.

The precision of our search was further enhanced by manually eliminating irrelevant terms and consolidating synonyms under the keyword plus category. This was followed by a meticulous manual filtering process to remove extraneous items from our search results. The final dataset was then subjected to analytical processing using R, VOSviewer, and CiteSpace6.3.1.0. Our paper presents a multifaceted network analysis based on the literature data we collected. This includes a co-citation network analysis of references in papers addressing drought and salt stresses in oat and a cooperative network analysis encompassing national networks. Additionally, we conducted a co-occurrence network analysis focusing on ‘keywords plus,’ as well as a coupling network analysis that includes institutional affiliations.

**Figure 9:**
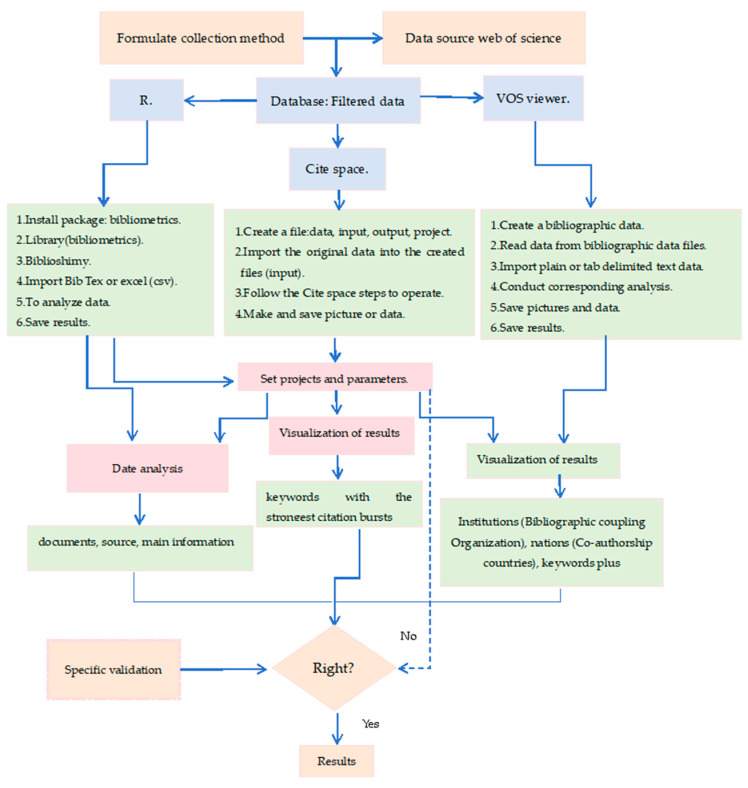
R and VOSviewer and Citespace bibliometric analysis flowchart.

The methodologies and procedural steps employed in using the corresponding software tools are illustrated in Figure 9. This comprehensive approach enabled us to systematically analyze and interpret the vast array of data pertaining to the research on oat under the duress of drought and salt stresses, offering insightful perspectives into the current state of research and potential future directions in this field.

## 5. Conclusions

This bibliometric study comprehensively examined oat’s responses to drought and salt stresses from 1993 to 2023. It identified a steady increase in annual publications on drought stress in oat, totaling 224 over the past decade. With contributions from 585 institutions across 69 countries, notable institutions like the Agricultural University of Canada and China Agricultural University led the research. The U.S., Canada, and China emerged as top contributors. Collaboration among these countries has notably advanced the field, with research mainly published in field crop journals. Notably, papers cited in the Proceedings of the National Academy of Sciences of the USA underscore the global significance of this research. Studies mainly focused on physiological, biochemical, morphological, and genetic aspects of drought resilience, hinting at future trends in gene and protein research for drought resistance. Similarly, research on oat’s response to salt stress saw an upward trend, with 334 papers published. Leading institutions like the Chinese Academy of Agricultural Sciences and Inner Mongolia Agricultural University played significant roles. China, the U.S., and India led in publication volume, emphasizing their influence. Research primarily addressed growth, development, yield variability, and physiological responses to salt stress, providing a scientific basis for enhancing oat adaptability and yield. Future directions may include advanced gene-editing technologies and detailed physiological studies to uncover new salt-tolerance mechanisms and breed resilient varieties. Despite achievements under individual stress conditions, combined drought and salt-stress effects on oat remain understudied, necessitating future research on their interaction at various biological levels. This review offers vital insights into plant adaptation strategies, guiding future agricultural interventions to boost crop resilience and productivity in challenging conditions.

## Figures and Tables

**Table 1:** The top 10 organizations in the total amount of drought-stress-related research published in oat in the past 30 years (1993–2023).

Ranking	Institutions	TP	TLS	TC	Country
1	Canadian Agricultural Food University	19	1512	335	Canada
2	China Agricultural University	15	1627	183	China
3	CSS Heavy Industry Co. Ltd	14	1201	330	China
4	Agricultural Science of China Academy of Sciences	13	949	137	China
5	Inner Mongolia Agricultural University	12	1119	115	China
6	Lanzhou University	10	695	112	China
7	USDA-ARS	8	41	189	USA
8	Aberystwyth University	7	1062	234	Britain
9	Baocheng academic Agricultural Science	7	1006	103	China
10	Polish Academy of Sciences	7	480	41	Poland

TP is total papers; TLS is total link strength; TC is total citations.

**Table 2:** The top 10 organizations in the total amount of salt-stress-related research published in oat in the past 30 years (1993–2023).

Ranking	Institutions	TP	TLS	TC	Country
1	Agricultural Science of China Academy of Sciences	21	2036	215	China
2	Inner Mongolia Agricultural University	19	2171	187	China
3	Canadian agricultural Food University	9	728	386	Canada
4	China Agricultural University	8	946	125	China
5	Gansu Agricultural University	5	647	231	China
6	Yangzhou University	5	230	42	China
7	Bose Inst	4	238	289	USA
8	CSS Heavy Industry Coltd	4	162	183	China
9	Lanzhou University	4	322	103	China
10	South China Agricultural University	4	285	31	China

TP is total papers; TLS is total link strength; TC is total citations.

**Table 3:** The top 10 countries in the total amount of drought-stress-related research published in oat in the past 30 years (1993–2023).

Ranking	Country	TP	TC	AC
1	China	91	1211	13.31
2	USA	63	2034	32.29
3	Canada	36	646	17.94
4	Australia	28	649	23.18
5	Spain	23	458	19.9
6	India	19	603	31.74
7	England	17	312	18.35
8	Poland	16	61	3.81
9	Germany	14	572	40.36
10	France	12	378	31.5

TP is total papers; TC is total citations; AC is average citation.

**Table 4:** The top 10 countries in the total amount of salt-stress-related research published in oat in the past 30 years (1993–2023).

Ranking	Country	TP	TC	AC
1	China	97	1676	17.28
2	USA	45	2337	51.93
3	India	33	1190	36.06
4	Canada	18	584	32.44
5	Australia	13	215	16.54
6	Spain	12	751	62.58
7	Pakistan	10	122	12.20
8	England	9	463	51.44
9	Iran	9	149	16.56
10	Poland	9	175	19.44

TP is total papers; TC is total citations; AC is average citation.

**Table 5:** The most relevant source information to oat to drought stress.

Ranking	Element	H_Index	TC	NP	IF/Year
1	*Proceedings of the National Academy of Sciences of the United States of America*	2	942	2	11.1 2020–2021
2	*Physiologia Plantarum*	7	711	7	6.4 2022–2023
3	*Field Crops Research*	12	480	16	5.8 2022–2023
4	*Agricultural and Forest Meteorology*	2	300	2	6.2 2021–2022
5	*Plant Science*	4	240	4	4.3 2020–2021
6	*Plant Physiology*	2	231	2	7.4 2022–2023
7	*Plant Cell and Environment*	3	230	3	7.3 2022–2023
8	*Photosynthetica*	2	226	2	2.7 2022–2023
9	*Ecological Applications*	1	184	1	5.0 2022–2023
10	*Canadian journal of Plant Science*	6	170	9	1.2 2022–2023

TC is total citations; NP is Number of publications; IF is the impact factor.

**Table 6:** The most relevant source information to oat to salt stress.

Ranking	Element	H_Index	TC	NP	IF/Year
1	*Plant Science*	5	1245	5	4.3 2020–2021
2	*Physiologia Plantarum*	8	859	8	6.4 2022–2023
3	*Proceedings of the National Academy of Sciences of the United States of America*	1	529	1	11.1 2020–2021
4	*Plant Growth Regulation*	7	365	7	4.2 2022–2023
5	*Plant Physiology*	3	333	3	7.4 2022–2023
6	*Journal of Nutrition*	3	259	4	4.2 2016–2017
7	*Plant Physiology and Biochemistry*	7	253	8	6.5 2022–2023
8	*Photosynthetica*	1	220	1	2.7 2022–2023
9	*Plant Journal*	2	213	2	7.2 2022–2023
10	*Plant Cell and Environment*	3	211	3	7.3 2022–2023

TC is total citations; NP is Number of publications; IF is the impact factor.

**Table 9:** Top 15 keywords in centrality (1993–2023).

Drought Stress			Salt Stress		
Keywords	Centrality	Count	Keywords	Centrality	Count
Drought stress	0.72	280	Salt stress	0.64	180
Oats	0.4	60	Avena staiva	0.26	34
Abiotic stress	0.26	81	abiotic stress	0.2	48
Growth	0.24	77	Abscisic acid	0.14	15
Yield	0.24	60	Arginine decarboxylase	0.13	15
Abscisic acid	0.12	22	Growth	1	47
Climate change	0.1	25	Biosynthesis	0.09	20
Photosynthesis	0.1	17	Oxidative stress	0.08	16
Arabidopsis	0.08	15	Gene	0.08	12
Cultivars	0.07	12	Arabidopsis	0.08	15
Gene	0.06	16	Proline	0.07	14
Leaves	0.06	12	Bacteria	0.07	6
Oxidative stress	0.05	14	Gene expression	0.05	8
Temperature	0.04	14	Metabolism	0.05	10
Quality	0.04	17	Alkali stress	0.05	8

## Data Availability

The original contributions presented in the study are included in the article, further inquiries can be directed to the corresponding authors.
